# 349. Characteristics and Outcomes of COVID-19 Patients with Candidemia at a Community Teaching Hospital in Chicago - One Year Follow Up.

**DOI:** 10.1093/ofid/ofac492.427

**Published:** 2022-12-15

**Authors:** Oluwadamilola A Adeyemi, David Okoh, Gregg Gonzaga, Sean Cariño, Steve B Kalish

**Affiliations:** Swedish Hospital Part of NorthShore University HealthSystem, Chicago, Illinois; University of Birmingham, Birmingham, England, United Kingdom; Swedish Hospital Part of NorthShore University HealthSystem, Chicago, Illinois; Swedish Hospital Part of NorthShore University HealthSystem, Chicago, Illinois; Swedish Hospital Part of NorthShore University HealthSystem, Chicago, Illinois

## Abstract

**Background:**

We previously reported an alarming increase in cases of nosocomial Candidemia at our hospital which were associated with acute COVID-19 infection (Abstract 287 IDWeek 2021). We reinstated mitigation strategies including staff education, line insertion check list and antimicrobial stewardship. 1041 patients with acute COVID-19 were admitted to our hospital in 2021 (January to December) and 6 out 12 cases (50%) of Nosocomial Candidemia were seen in patients with acute COVID-19 infection. We re-evaluated the risk factors and mortality of hospitalized COVID-19 patients with Candidemia.

**Methods:**

We performed a retrospective chart review of the 6 patients with Candidemia and confirmed COVID-19 infection at our 292-bed community teaching hospital in Chicago, Illinois from January through December 2021. We report a descriptive analysis of the demographic characteristics, comorbidities, complications, and outcomes of these patients comparing both years.

**Results:**

The average age of our study population was 71 years (older); 67% were male. The average hospital length of stay (LOS) was shorter 28 days. The mean time from admission to the development of Candidemia was slightly longer 18 days. Associated co-morbidities included cardiovascular diseases (CVD) in 83%, diabetes mellitus (DM), in 50%, and obesity in 50%. Treatments for COVID-19 included Steroids (100%), Remdesivir (50%) and Baricitinib (33%). All patients were managed in the intensive care unit (ICU) and 67% had a central in place at the time of Candidemia. Half of the patients (50%) required hemodialysis (HD); all patients were treated with multiple antibiotics. The average LOS in the ICU was 18 days (shorter). Despite antifungal treatment, 80% expired.

**Conclusion:**

Incidence of Candidemia in acute COVID-19 infections decreased by 56% in one year after reinstating mitigation strategies in our hospital. However, Candidemia remains a menace in hospitalized patients with acute COVID-19 infection. Associated risk factors remain history of CVD, DM, obesity, prolonged hospital LOS, requirement for multiple CL, HD, treatment with multiple antibiotics, treatment with steroids (100%) and a long stay in the ICU. The mortality of COVID-19 patients with Candidemia remains very high.

**Disclosures:**

**All Authors**: No reported disclosures.

